# Vocal Feature Extraction-Based Artificial Intelligent Model for Parkinson’s Disease Detection

**DOI:** 10.3390/diagnostics11061076

**Published:** 2021-06-11

**Authors:** Muntasir Hoq, Mohammed Nazim Uddin, Seung-Bo Park

**Affiliations:** 1 Department of Computer Science and Engineering, East Delta University, Chattogram 4209, Bangladesh; muntasir.h@eastdelta.edu.bd; 2 Department of Software Convergence Engineering, Inha University, Incheon 22201, Korea; molaal@inha.ac.kr

**Keywords:** medical analytics, Parkinson’s disease detection, principal component analysis, sparse autoencoder, support vector machine

## Abstract

As a neurodegenerative disorder, Parkinson’s disease (PD) affects the nerve cells of the human brain. Early detection and treatment can help to relieve the symptoms of PD. Recent PD studies have extracted the features from vocal disorders as a harbinger for PD detection, as patients face vocal changes and impairments at the early stages of PD. In this study, two hybrid models based on a Support Vector Machine (SVM) integrating with a Principal Component Analysis (PCA) and a Sparse Autoencoder (SAE) are proposed to detect PD patients based on their vocal features. The first model extracted and reduced the principal components of vocal features based on the explained variance of each feature using PCA. For the first time, the second model used a novel Deep Neural Network (DNN) of an SAE, consisting of multiple hidden layers with L1 regularization to compress the vocal features into lower-dimensional latent space. In both models, reduced features were fed into the SVM as inputs, which performed classification by learning hyperplanes, along with projecting the data into a higher dimension. An F1-score, a Mathews Correlation Coefficient (MCC), and a Precision-Recall curve were used, along with accuracy to evaluate the proposed models due to highly imbalanced data. With its highest accuracy of 0.935, F1-score of 0.951, and MCC value of 0.788, the probing results show that the proposed model of the SAE-SVM surpassed not only the former model of the PCA-SVM and other standard models including Multilayer Perceptron (MLP), Extreme Gradient Boosting (XGBoost), K-Nearest Neighbor (KNN), and Random Forest (RF), but also surpassed two recent studies using the same dataset. Oversampling and balancing the dataset with SMOTE boosted the performance of the models.

## 1. Introduction

Parkinson’s disease (PD), a long-term neurodegenerative disorder affecting the human motor system, results in many motor and non-motor characteristics [1]. It is considered one of the most common movement disorders among individuals over 60 years of age [2]. PD has a Relative Risk (RR) of death of 2.3 (1.8 to 3.0) [3]. PD can be detected by observing changes in behavioral patterns, as well as by observing rigidity, cognitive impairment, bradykinesia, tremors, and postural instability [4]. PD is currently incurable, but treatment following early diagnosis can improve and relieve the symptoms. Most people experience a normal life expectancy of another 7 to 14 years after diagnosing PD [5]. However, PD has a more than 25% rate of misdiagnosis [6]. More accurate detection of PD can increase the life expectancy for a diagnosed PD patient by preventing complications and maintaining a high quality of life with the necessary pharmacological and surgical intervention [7].

In order to detect PD at an early stage, many health informatics systems, including telediagnosis and telemonitoring systems, have been developed for current pharmacological therapeutics [8]. Researchers are concentrating on identifying biological markers for the detection phase. At present, the application of Machine Learning (ML) is thriving in the field of prediction; hence, it is widely used in PD diagnosis. Neuroimaging modalities, including feature extraction from the processing of scanned data such as MRI images using different ML techniques, are paving a promising way towards detecting PD [9,10,11]. These health informatics systems aim to reduce the discommoding physical visits to clinics for clinical examination, which, as a result, will reduce the task of health workers and clinicians [12,13,14,15].

There are many symptoms that appear in PD patients, including posture and balance deficiencies, dysphonia (change in speech and articulation), and slowed movement, etc. Among these symptoms is vocal dysfunction, which results in vocal instability, loudness, and damaged vocal quality. Therefore, the early detection of PD can be done by analyzing speech signals, as 90% of PD patients face vocal problems in the incipient stage of the disease [16]. Consequently, the recent focus of PD detection research emphasizes the vocal disorders of patients. In recent studies, clinical features from the speech of PD patients were extracted and fed into a classification model with the help of various speech signal processing algorithms. Based on speech recordings, this telemonitoring study mapped the vocal features of PD to a clinical evaluation system that predicted the possibility of PD in patients. Moreover, the collection of speech data was a non-invasive process, which made the data easy to collect and subsequently provide as the input of the telediagnosis system.

ML techniques including Artificial Neural Networks (ANN) [17], K-Nearest Neighbors (KNN) [18], Random Forest (RF) [19], and Extreme Gradient Boosting (XGBoost) [20] have been used in PD classification based on patient vocal disorders. However, the success rate of accurate detection depends on the quality of data, on the relevance of the features extracted from them, and on the associated ML models. Many recent studies have been conducted on a publicly available dataset that consists of the sound measurements of 8 healthy and 23 PD-affected instances, aggregating 195 data samples [21]. Another publicly available dataset includes the data of 20 PD patients and 20 healthy individuals [12]. Both datasets consist of some common features extracted from the speech signals, including vocal fundamental frequency, measures of the ratio of the noise-to-tonal components, measures of variation in amplitude, measures of variation in fundamental frequency, etc. Since a good number of the studies regarding PD classification from vocal features are conducted with these datasets, the features extracted from these datasets are referred to as the baseline features. Other features are also used to detect PD, such as Mel-frequency Cepstral Coefficients (MFCC) and Signal-to-Noise Ratio (SNR) [22]. In a recent study, the effectiveness of vocal features was analyzed [15]. Although slow movement, tremors, inertia, and balance deficiency are among the symptoms of PD, vocal and speech signal processing is widely used as they can be easily tracked by the changes of speech along with other symptoms’ data, as formerly stated, from wearable sensors.

Sharma and Giri extracted different features from voice signals, including MFCC, jitter, shimmer features, pitch, and glottal pulse [23]. These feature values of a PD patient show higher fluctuations and variance than those of a normal person. Tunable Q-factor Wavelet Transform (TQWT) was introduced in a recent study by Sakar et al. [16], along with other features, to detect PD patients from their vocal signals. The same dataset was being experimented in a study using Deep Learning techniques, where two frameworks consisting of Convolutional Neural Networks (CNN) were applied [24]. However, properly training Deep Neural Networks (DNN) to converge requires very large datasets and also takes substantial training time in order to search the parameter space. In addition, dividing all the features into different sets, a feature that is more relevant and important in the classification process, is treated in the same way as other less important features of the same set, which decreases the accuracy of detecting a PD patient.

Bouchikhi et al. [25] proposed a model with an SVM as a classifier and Relief-F as a feature selection method. In their study, the feature set was reduced from 22 to 10. Subsequently, the SVM classifier with a 10-fold cross-validation method showed that Relief-F had an accuracy rate of 96.88%. The dataset contained 195 voice samples. However, it was empirically and theoretically proved that the performance of core relief-based algorithms (RBA) decreases drastically as the number of irrelevant features becomes enormous [26]. This is because as the number of features increases, Relief’s computation of neighbors and weights becomes excessively random, which is an example of curse dimensionality. Additionally, [27] found that RBAs are vulnerable to noise interfering with the section of nearest neighbors.

Hemmerling and Sztahó [28], on the other hand, proposed the use of PCA with a non-linear SVM to identify PD patients, and their methods show a classification accuracy rate of 93.43%. However, the dataset used in their study was comparatively small. Small datasets have several disadvantages, leading to lower precision in the prediction and lower power. They also pose a greater risk as they may compare the classes unfairly, even in circumstances where the data is from a randomized trial.

Our study adopted two different feature extraction techniques, including Principal Component Analysis (PCA) [29] and a novel deeper Sparse Autoencoder (SAE) [30], where the PCA is widely used when the features are linearly related and our novel SAE is capable of working well with non-linearly related features in order to reduce the feature set dimensionality and take into account the most important and relevant information for classification. Moreover, feeding the model irrelevant and high dimensional data risks over-fitting. In order to classify between a PD patient and a healthy individual, the Support Vector Machine [31,32] is employed as a powerful classification method that exceeds other techniques with fewer subjects. When the number of features is high, SVM is more efficient at classification task in high dimensional spaces with less training data, delivering a unique solution surpassing the Neural Networks in the case of convex optimality problems. This study aimed to eliminate the drawbacks of both Gunduz [24] and Sakar et al. [16] by applying two hybrid models integrating PCA-SVM and SAE-SVM.

The rest of the paper is organized as follows: Section 2 summarizes the related work conducted in this domain, Section 3 contains the description of the dataset, Section 4 describes the methodologies that are used in this study, Section 5 states the experimental results and comparison with different traditional and state-of-the-art disciplines, and Section 6 briefly draws the findings and future directions of research.

## 2. Related Works

In this section, various recent studies on PD classification using machine learning algorithms and deep learning methods have been summarized.

In [33], Karimi Rouzbahani and Daliri used voice signals for PD detection. Parameters such as pitch, jitter, fundamental frequency, shimmer, and various statistical measures based on these parameters were used as the input of the proposed predictive model. The harmonics-to-noise ratio and the noise-to-harmonics ratio were also extracted using estimates of signal-to-noise by calculation of the autocorrelation of each cycle. In their study, one of every two features which resulted in a correlation rate of over 95% was removed. Several feature selection methods, such as Receiver Operating Characteristics (ROC) curves, *t*-test, and Fisher’s Discriminant Ratio (FDR), were utilized to identify the informative features among the whole feature set. The number of features was incremented one by one. Afterwards, the prioritized features were fed into an SVM classifier. The highest performance was achieved using three classifiers at hand and a combination of the seven most prior features. The selected features were used to train the SVM, KNN, and discrimination-function-based (DBF) classifiers. Among these classifiers, KNN showed the best performance with an accuracy rate of 93.82%. KNN has also shown good performance in other performance matrices such as error rate, sensitivity, and specificity.

Ma et al. [34] proposed a novel hybrid method integrating subtractive clustering features weighting (SCFW) and kernel-based extreme learning machine (KELM) for the diagnosis of PD patients. SCFW, a data-preprocessing tool, is used to decrease the variance in the dataset. The output of the SCFW further improved the accuracy of the KELM classifier. The efficiency of the proposed method was justified in terms of accuracy, specificity, sensitivity, Area under the ROC Curve (AUC), kappa statistic value, and f-measure. The proposed method outperformed (via a 10-fold cross-validation scheme) the SVM-based, ELM-based, KNN-based, and other methods with a classification accuracy of 99.49%.

Zuo et al. [35] presented an effective method for the diagnosis of PD patients using particle swarm optimization (PSO) enhanced fuzzy k-nearest neighbor (FKNN). PSO-FKNN uses both the continuous and binary versions of PSO to achieve parameter optimization and feature selection concurrently. The continuous PSO adaptively specifies the fuzzy strength parameter m in and the neighborhood size k in FKNN, whereas the most discerning subset of features in the dataset is chosen by the binary PSO. This PSO-FKNN model was justified in terms of the accuracy, specificity, sensitivity, and the AUC of the ROC curve. The proposed model achieved a mean accuracy rate of 97.47% via a 10-fold cross-validation analysis.

Sharma and Giri [23] applied three types of classifiers based on MLP, KNN, and SVM to diagnose PD patients. Among these, SVM with an RBF kernel showed the best result with a total classification accuracy rate of 85.294%.

Parisi et al. [36] proposed an artificial intelligent classifier using Multilayer Perceptron (MLP) and Lagrangian Support Vector Machine (LSVM). The importance scores of the features were assigned by the MLP with custom cost functions, which included both the AUC score and the accuracy. The MLP provided the 20 most important features with high importance scores, which were then fed into the LSVM classifier. The proposed method achieved an accuracy rate of 100% and was compared with other similar studies.

For the first time, Sakar et al. [16] introduced tunable Q-factor wavelet transform (TQWT) to vocal signals for the diagnosis of PD patients. The feature subsets obtained from the dataset were given as input in various classifiers, and the study showed that TQWT-based features tend to achieve better results than the other popular voice features used in PD classification. In their study, a combination of TQWT plus Mel-frequency cepstral coefficients (MFCC) plus Concat showed the best performance in terms of all metrics and among all classifiers with an accuracy rate of 85.7%, an F1-score of 0.84, and an MCC value of 0.59.

Gunduz [24] used the same dataset used in Sakar et al. [16] to diagnose PD patients using Deep Learning Techniques, where two frameworks consisting of CNN were applied. Both of the studies used the features as subsets and estimated the accuracy by a combination of input into the ML models. Gunduz [24] showed a higher accuracy of 86.9%, an F1-score of 0.910, and an MCC value of 0.632.

Caliskan et al. [37] proposed a PD detection model, which is a DNN classifier consisting of a stacked autoencoder used for obtaining inherent information within the vocal features. Their proposed model was compared with several other state-of-the-art ML models, and it was concluded that the DNN classifier was convenient in the diagnosis of PD patients. However, DNN requires plenty of data to be suitably trained to converge and also takes a lot of training time in order to search the parameter space. Moreover, their study focused only on feature extraction by redundancy removal while classifying PD patients.

In another study, Wroge et al. [38] performed the diagnosis of PD patients using DNN. A mobile application was used to collect the data of PD patients and non-PD patients. Two types of feature sets were obtained from the collected data. The first one was the Audio-Visual Emotion recognition Challenge (AVEC), which had dimensions up to 2200, and the second features set contained 60 features that were set up using MFCC. The features were given as input in a three-layered DNN and other AI classifiers. The results showed that DNN had the highest accuracy rate of 85% compared to the average clinical diagnosis accuracy rate of 73.8%.

The above-mentioned studies are based on vocal features as an important factor for detecting PD patients. Apart from these, several other studies have also been performed which extracts features from different datasets, e.g., wearable sensors [39], electroencephalogram (EEG) [40], and smart pens [41].

## 3. Dataset

The dataset was obtained from the University of California-Irvine (UCI) Machine Learning repository and was used in [16,24] before the present study. Table 1 contains the details of the dataset. The dataset has an imbalance regarding the number of men and women (a ratio of 23:41) and healthy individuals and PD patients (a ratio of 107:81). PD is 1.5 times more common in men, and along with that, motor progression is more aggressive in men than in women. Nevertheless, there are no significant differences in terms of demographic variables [42].

As speech anomalies are one of the key effects that have been seen in PD patients, vocal features and speech signal attributes have been used successfully to assess PD. The traditional features mostly used in PD detection are the fundamental frequency parameters, Recurrence Period Density Entropy (RPDE), jitter, harmonicity parameters, Pitch Period Entropy (PPE), Detrended Fluctuation Analysis (DFA), etc. [14,15,21,22,43]. In [16], these features were classified as baseline features. Additionally, Praat acoustic analysis software [44] was used to extract these baseline features. The description of the features is provided in Table 2. Feature engineering is adopted from [16] in our study.

**Figure 1:**
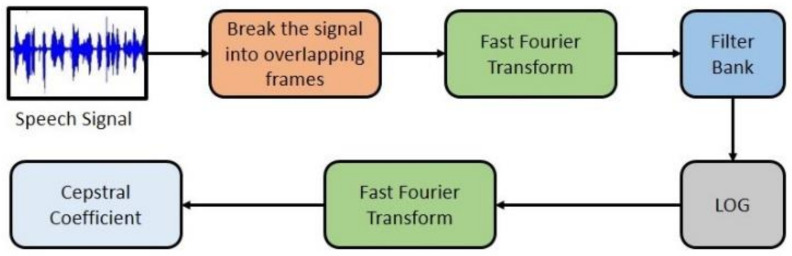
Mel-Frequency Cepstral Coefficients (MFCC) block diagram.

The shape of the human vocal tracts (e.g., teeth, tongue etc.) is the most important component of any sound generation. To accurately represent this sound, this shape must be determined correctly. The vocal tract representative is the envelope of the time power spectrum. This envelope is accurately represented by Mel-Frequency Cepstral Coefficients (MFCCs). In other words, MFCCs can imitate the characteristics of the human ear and have been used in different speech recognition tasks [45,46]. In this study, MFCCs are being employed to detect the aberration in the human tongue and lips, which are directly affected by PD. In Figure 1, a stepwise summary of the MFCC block diagram is shown.

The formula for the frequency to Mel scale is given below:

(1) $$M\left(f\right)=1125\ln\left(1+\frac{f}{700}\right)$$

where *f* = frequency of the signal.

The Mel-scale relates the acquired frequency of a tone to the actual measured frequency, which scales the frequency to mimic the human ear. Cepstrum is the information rate of change in spectral bands. Mel frequency cepstral is obtained by taking the log of the magnitude of the Fourier spectrum and later taking the spectrum of this log by a cosine transformation; there is a peak observed where there is a periodic element in the original time signal. Upon applying a transform on the frequency spectrum, the resulting spectrum is in neither the time domain nor the frequency domain. Hence, it is called the “quefrency domain” [47]. The log of the spectrum of the time signal was named cepstrum.

Wavelet transform (WT), similar to Fourier transform, was used with a completely different merit function that uses functions which are confined in both real and Fourier space. The following equation expresses the mathematical function of WT:

(2) $$F\left(a,b\right)=\int_{-\infty }^{+\infty }f\left(x\right)\varphi_{\left(a,b\right)}^{*}\left(x\right)dx$$

where φ= some function and * is the complex conjugate symbol.

From Equation (2), it can be inferred that WT is an infinite set of various transforms, depending on the merit function used for its computation. It can be useful for making decisions on a signal, especially on the regional scale with small fluctuations. WT is a very popular tool as, in several studies, special features have been extracted from the basic frequency of the speech signal (F0). Speech sample deviation can be captured by WT-based features [48]. This is how WT can detect sudden aberrations of long-term vowels in clinical speech recordings. In this dataset, the WT-based feature number is 182, which includes the Shannon’s and the log energy entropy, the energy, and the Teager-Kaiser energy of both the approximation and detailed coefficients. WT-based features obtained from the raw (F0) contour and the log transformation of the (F0) contour have been collected using a 10-level discrete wavelet transform.

In a very recent work [16], TQWT-based features were used. It is a completely discrete, over-complete WT and the main feature extractor [49]. To transform signals in better quality, TQWT uses three tunable parameters, which are Q-factor (Q), redundancy (r), and the number of levels (J). Speech signals have high oscillatory time series characteristics for which a Q-factor with a relatively high value is appropriate. The TQWT consists of two filter banks. The low pass filter (LPF) output is provided as the inputs of the second LPF or the high pass filter (HPF) bank. The filter banks are iteratively applied. If J is the number of levels, J HPF and one final LPF output will provide J + 1 sub-bands at the end of the decomposition stage. The redundancy rate (r), also known as the decomposition rate, controls the unexpected excessive ringing. Without affecting the shape, this process helps to localize the wavelets in the time domain [49]. The TQWT parameters are determined in the following order:

- Defining the Q-factor parameter to regulate the oscillatory behavior of the wavelet.
- Setting the r parameter value greater or equal to three to prevent the undesired ringing in wavelets.
- Searching for the best accuracy value in different Q–r pairs of several numbers of levels (J) in the fixed intervals.

There are a total of 432 TQWT-related features available in this data set.

Apart from the aforementioned features, several other features have also been employed depending on vocal fold vibration. Features such as the Vocal Fold Excitation Ratio (VFER), Glottal to Noise Excitation (GNE), the Glottis Quotient (GQ), etc., have also been employed to explore the effect of noise on the vocal fold.

To be mentioned, min-max normalization on this data has been performed to keep the same differences in the range of values by fitting the feature values into a common scale. The normalization process is a data pre-processing part to handle the bias to larger feature values [50].

## 4. Methodologies

### 4.1. Principal Component Analysis (PCA)

A large number of features and high dimensional data increase computational costs, memory usage and also reduce accuracy. Principal Component Analysis (PCA) orthogonally transforms sets of correlated variables into sets of linearly uncorrelated ones [51]. These new uncorrelated features are called the principal components and are equal or lesser in number than the original variables.

In PCA, the covariance matrix of the data points X is calculated, where X is a m×n matrix, m being the dimension and *n* being the number of data points. The Eigenvectors are sorted in descending order according to the Eigenvalues calculated. The first k Eigenvectors are chosen if the number of principal components corresponds to k. The covariance matrix $C_{x}$ can be calculated by Equation (3):

(3) $$C_{x}=\frac{1}{n-1}\left(X-\bar{X}\right){\left(X-\bar{X}\right)}^{T}$$

where $\bar{X}$ = Transpose of X.

PCA is an unsupervised technique without knowing the actual class labels to which the features belong. It is the most effective technique in extracting the most relevant and important information from redundant and noisy data. The principal components will be independent if the data comes from a normal distribution. Through PCA, it is expected that the principal components will cover the maximum variations of the data and thus effectively reduce the data dimension. The dimensionality of the data is reduced by projecting them onto a lower dimension [52]. The first principal component is chosen to reduce the distance between the data and the projection. The subsequent components are also selected similarly but are uncorrelated with the previous principal components. As a result, the data dimension is reduced by eliminating the weaker components and removing redundant information [53].

### 4.2. Sparse Autoencoder (SAE)

**Figure 2:**
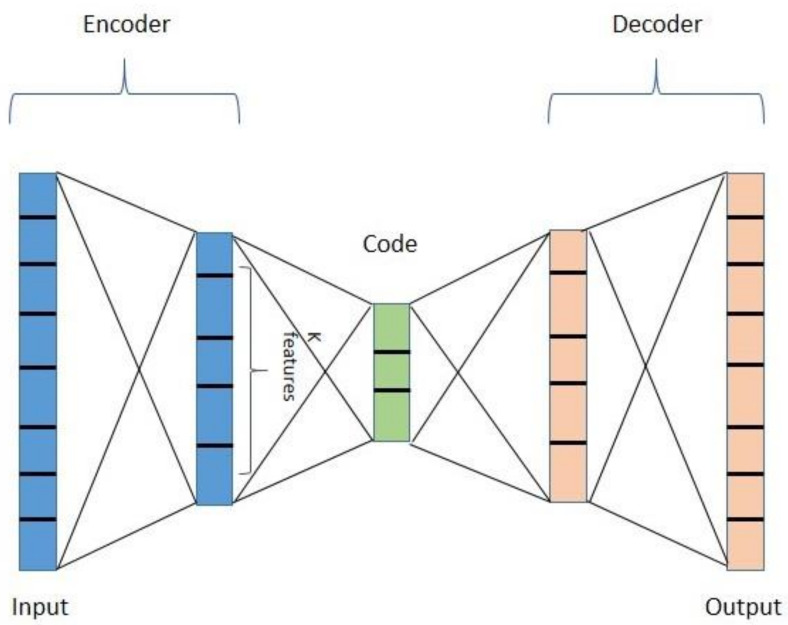
An Autoencoder with two hidden layers.

An Autoencoder is an axisymmetric single hidden-layer neural network [54]. It is an unsupervised technique for feature extraction using DNN. Using the hidden layer, the Autoencoder encodes the input data, estimates the minimum error, and collects the best-feature hidden-layer expression [55]. The unsupervised computational simulation of human intuitive learning is the Autoencoder concept’s driving factor, which has some functional flaws [56]. Although the Autoencoder can reconstruct input data with high precision, it cannot learn any practical features through copying and inputting memory into an implicit layer. This is where the idea of SAE comes, which inherits the principle of Autoencoder and introduces the sparse penalty term, also known as sparsity regularization. It adds restrictions to feature learning for a concise expression of the input data [57]. Equation (7) depicts the sparsity regularization term for the SAE. Here, the process is divided into three parts: encoder, code, and decoder. The encoder part encodes the inputs into code via hidden layers, and the decoder decodes the code into the output layer, where outputs are the same as the inputs. The code is a compression and is also known as the latent space representation. Figure 2 shows different parts of an Autoencoder, where the encoder maps the inputs to k features. The Sigmoid activation function and the Rectified Linear Unit (ReLU) are commonly used for the non-linear mapping of the Autoencoder. The ReLU in Autoencoders has a huge drawback of dealing with negative values becoming zero, which eventually decreases the ability to train the model properly. In the case of the vocal features of PD patients, it is a concern as the dataset used in this study contains negative feature values along with positive ones. The objective of the Autoencoders is to learn features from the inputs, which can be reconstructed by learning the encoding and decoding functions and minimizing the error between the inputs and the reconstructed data. The SAE can be regularized by involving a sparsity constraint, which ensures that only a few nodes are active and penalizes the hidden layers. In the feature extraction process, L1 regularization or Lasso regression was used in this study as it compresses features by treating the less important feature coefficients as zero, thus shrinking the parameter space. Equation (6) states the mathematical formulation of L1 regularization. Regularization results in avoiding overfitting and thus performing well on new examples. After learning the features from the Autoencoder, the encoder part can be merged with a classifier to classify data points.

Here, Mean Squared Error (*MSE*) is used for this task which can be defined as:

(4) $$MSE=\frac{1}{N}{\left|X-\hat{X}\right|}^{2}$$

where, X is input, $\hat{X}$ is the reconstructed output, and *N* is the total number of data points.

Equation (5) depicts the cost function for training an SAE, which includes three terms. The first term in *MSE* gives the discrepancy between input X and reconstructed $\hat{X}$ over the whole training dataset [30].

E = *MSE* + λ × L1 regularization term + β × sparsity regularization term (5)

where, λ is the coefficient of the L1 regularization and β is the coefficient of the sparsity regularization. The L1 regularization adds the “absolute value of magnitude” of the coefficients as a penalty term.

(6) $$L1\;\mathrm{regularization}\;\mathrm{term}=\sum_{l=1}^{n_{l}-1}\sum_{i=1}^{s_{l}}\sum_{j=1}^{s_{l}+1}\left|w_{ji}^{\left(l\right)}\right|$$

where *n_l_* = number of layers, *l* = Layer l, *s_l_* = number of units in *l* layer, and *w_ji_*^(*l*)^ is the weight value between node *i* in layer *l* and node *j* in layer *l* + 1.

Sparsity regularity term is defined as:

(7) $$\mathrm{Sparsity}\;\mathrm{regularity}\;\mathrm{term}=\sum_{j=1}^{s_{2}}KL\;(\;\rho \;\|{\hat{\rho }}_{j})$$

where *KL* is the Kullback-Leibler (*KL*) divergence and ${\hat{\rho }}_{j}$ = average activation of hidden node *j* and *ρ* is a sparsity parameter. The mathematical representation of *KL* is:

(8) $$KL\;(\;\rho \;\|{\hat{\rho }}_{j})=\left(\rho \right)\log\frac{\rho }{{\hat{\rho }}_{j}}+\left(1-\rho \right)\log(\frac{1-\rho }{1-{\hat{\rho }}_{j}})$$

To make the learned representation more suitable for classification, an Autoencoder can be fine-tuned using labelled data after the completion of the unsupervised stage. It can be done by replacing its decoder layer with an output layer for label prediction. As the L1 regularization tends to shrink the penalty coefficient to zero, it works better for feature extraction.

Other popular feature extraction techniques are available such as Linear Discriminant Analysis (LDA), Independent Component Analysis (ICA), Kernel PCA (kPCA), etc. Although these are commonly used, they have some major drawbacks. ICA assumes that the independent components are non-Gaussian and statistically independent; it only separates the independent sources without compressing information. LDA assumes a normal distribution of features. It does not work well with a skewed dataset. In kernel PCA, an approximation is needed to calculate the k value. However, it becomes a major problem for a larger dataset. Our study tried to overcome these problems with simpler and faster PCA for linearly correlated features and with a novel deeper SAE to extract and reduce the dimensionality of non-linearly related feature space more efficiently.

### 4.3. Support Vector Machine (SVM)

A Support Vector Machine (SVM), a supervised algorithm, classifies data and also performs well for regression purposes. The SVM creates hyperplanes in order to separate data into class labels. It is mostly used for binary classification problems, similar to our study, where class 1 is labelled as a PD patient and class 0 for a healthy person.

(9) $$\gamma_{i}=\left\{1,\;0\right\}$$

where $\gamma_{i}$ is the set for binary class labels.

Therefore,

(10) $$x_{i}\;.\;w+b\geq +1,\;\gamma_{i}=1$$

(11) $$x_{i}\;.\;w+b\leq -1,\;\gamma_{i}=0$$

where, $x_{i}$ is the ith sample, w is normal to the hyperplane, and *b* is the distance of the hyperplane from the origin, which is called the bias.

**Figure 3:**
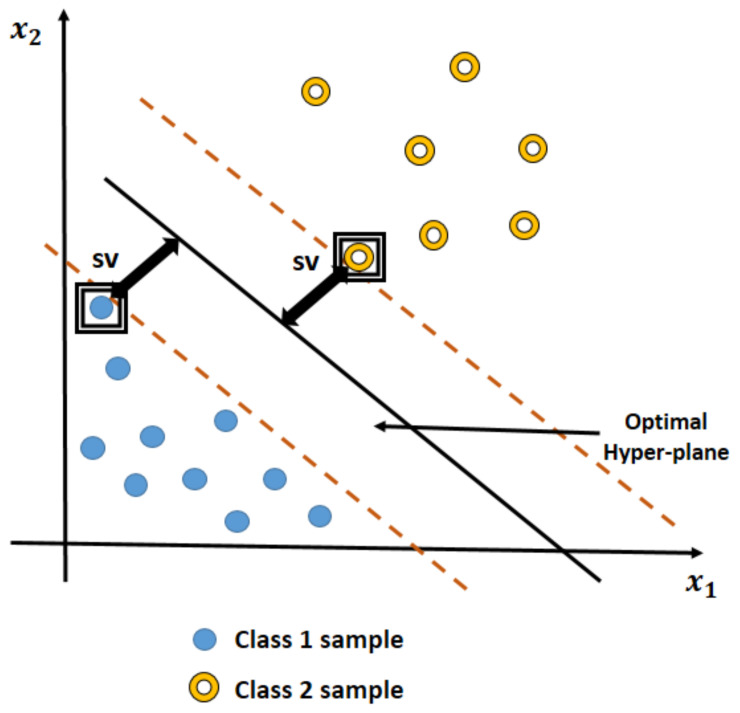
An SVM classifier with an optimal hyperplane.

The SVM classifier is based on the foundation of maximizing the hyperplane margin that classifies the data in the best way. Figure 3 shows an SVM classifier trained to separate data points in the N-dimensional space with support vectors (SV), which are the relevant data points lying on the margin boundaries. Here, N = 2 features ($x_{1},\;x_{2}$).

If the data points are linearly separable, then the data can be distinguished by hyperplanes which are infinite in number. SVM tries to find out the linear function having the largest margin that discriminates the class labels. In the SVM, with the help of kernel functions, input x is mapped onto a higher dimensional feature space. Then a linear model is built in this space. The common kernel functions of SVM are linear, radial basis function (RBF), polynomial, and sigmoid function. Via the kernel functions, the n samples are projected onto a new m dimensional space. The parameters of the SVM depend on the kernel function used. The regularization parameter for controlling the trade-off between misclassifications and the width of the margin in the SVM is C. A lower value of C causes under-fitting, and a higher value of C causes over-fitting of the model. The cost function for non-linear SVM, including the regularization parameter C, is expressed as:

(12) $$J\left(w\right)\;=C[\overset{m}{\underset{i=1}{\sum }}y^{\left(i\right)}Cost_{1}(w^{T}\left(f^{\left(i\right)}\right)+(1-y^{\left(i\right)})Cost_{0}(w^{T}(f^{\left(i\right)})]$$

where $J\left(w\right)$ is the cost function, w is normal to the hyperplane, C is the regularization parameter, $y^{\left(i\right)}$ is the class label for the *i*th sample, f is the kernel function, and the Cost function is defined as:

(13) $$Cost\left(h_{w}\left(x\right),y\right)=\left\{\begin{array}{c} \max\left(0,1-w^{T}x\right),\;if\;y=1\\ \max\left(0,1+w^{T}x\right),\;if\;y=0 \end{array}\right.$$

where x is the input sample for corresponding output label *y* and the SVM hypothesis $h_{w}\left(x\right)$ can be expressed as:

(14) $$h_{w}\left(x\right)=\left\{\begin{array}{c} 1,\;if\;w^{T}x\;\geq 0\\ 0,\;otherwise \end{array}\right.$$

These hyper-parameters can be set using k-fold cross-validation. This is used for evaluating ML models in a resampling procedure under limited data samples [58]. With the setting of the right kernel function and C value, SVM can be robust even if the data have some bias [59].

### 4.4. Proposed Model for PD Detection

As discussed earlier, SVM is effective and efficient in higher dimensional spaces. It is memory efficient, robust, and versatile, even when the number of dimensions is larger than the number of samples. However, if the number of features is excessively larger than the number of samples, the SVM’s performance will degrade radically. Thus, finding the most important features for the SVM using a PCA or SAE can outperform any other models. An SVM was used for PD detection in a few previous studies [24,60]. Yet, the lack of relevant feature extraction and removing redundant information led the SVM to perform poorly.

**Figure 4:**
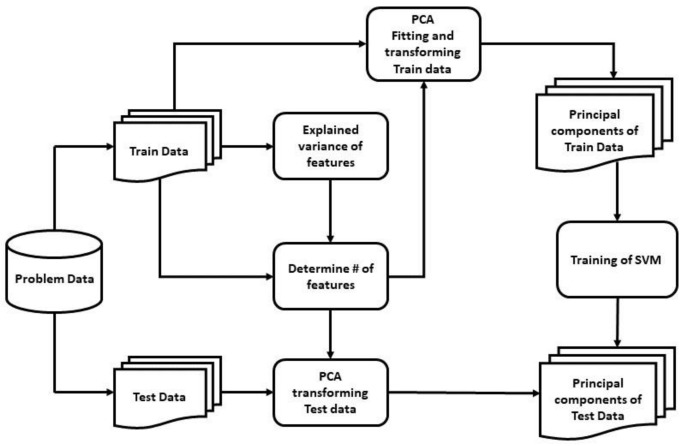
Flowchart of the PCA-SVM model.

**Figure 5:**
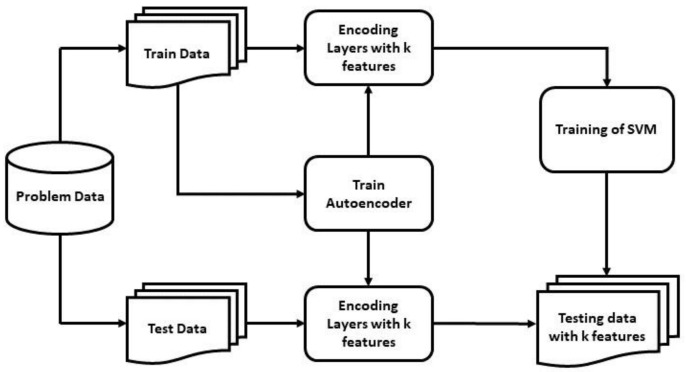
Flowchart of the SAE-SVM model.

In our study, two models for detecting PD patients were proposed using a PCA-SVM and an SAE-SVM hybridization by analyzing vocal disorders of patients. Figure 4 sketches the flowchart of the proposed model with the PCA-SVM, and Figure 5 represents the latter model integrating the SAE-SVM.

Learning all attributes and adding more dimensions can hinder rigorous model generalization. Hence, a PCA and a novel deeper SAE were used in our proposed models to reduce the number of dimensions. Generally, one hidden-layered SAE is used in feature compression in the field of PD detection [61]. However, our SAE-SVM model consisted of a deeper SAE with multiple hidden layers to improve the network’s generalization property, which is used for the first time as of our knowledge in vocal feature extraction to detect PD patients. In this study, we used ‘tanh’ activation function instead of the popularly used ReLU function for the non-linear mapping of the Autoencoder to prevent negative values from becoming zero, which could have decreased the ability to train the model properly. Above all, irrelevant and correlated features contribute the model to over-fit and thus decrease the performance in a real-world scenario. In the first model, we calculated the explained variance of each feature, which showed the proportion of which a model accounts for the dispersion or variance of the dataset [62]. The reduced number of principal components can be decided from the explained variance for better performance, and the reduced input dimension decreases the training time. These principal components were fed into the SVM to detect PD patients. In the second model, we trained a deep neural SAE, which is sparse in nature as L1 regularization was used. It mapped the input data into k feature space, and then these features were fed into the SVM to classify healthy individuals and PD patients.

In this study, we also applied the Synthetic Minority Over-sampling Technique (SMOTE) [63] to overcome the imbalanced characteristic of the dataset and have a limpid idea of the balanced-class performance of the models. Smote is a data augmentation technique. It synthesizes new examples from minority class (in this case, healthy patients) that are closer in the feature space. t-Distributed Stochastic Neighbor Embedding (t-SNE) was used to visualize the high-dimensional data [64]. t-SNE is another technique to reduce dimensionality and visualize high-dimensional data points in a probabilistic approach. It tries to measure the similarities in the high and low dimensional spaces between pair of examples and optimize these two similarities. With centering and measuring the density of all data points under Gaussian distribution, a set of probability for all points is obtained. These probabilities are proportional to the similarity measure. This means if two points have similar values, then they have local similarities in the high dimensional space. Perplexity is used to manipulate the distribution by influencing the variance of the distribution. Similarly, a Student t-distribution calculates the second set of probabilities in the low dimensional space with one degree of freedom. Finally, both the probability sets are used to measure the difference between the two-dimensional spaces.

The proposed methods were compared with each other, and the best one was compared with traditional ML techniques like Multilayer Perceptron (MLP), K-Nearest Neighbors (KNN), Random Forest (RF), and state-of-the-art disciplines such as Extreme Gradient Boosting (XGBoost). MLP, a Deep Artificial Neural Network, continuously updates weight matrices to minimize the error between actual values and predicted ones to find the optimal set of weights in the parameter space. It works well in non-linear classification problems but has the drawbacks of slow convergence rates and getting stuck at the local minima of the search space. KNN is a supervised learning algorithm, which means that it has to be provided with a labelled dataset [65]. It is also a non-parametric and instance-based algorithm. KNN runs through the whole dataset, computing the distance between an unseen observation and each training observation. Then, it estimates the conditional probability for each class in the set. However, KNN is computationally expensive, and it has a skewed class distribution problem. Moreover, the accuracy of KNN is downgraded with high-dimensional data. RF is an ensemble learning-based classification method [66]. It works on a technique called Bootstrap Aggregation, also known as bagging. Bagging involves training each decision tree on a different data sample, and the sampling is done with replacement. In summary, it combines multiple decision trees in determining the final output other than depending on each decision tree. However, overfitting, no interpretability, and poor performance in a large dataset are the drawbacks of RF. XGBoost is a model based on gradient boosted decision trees. It has outperformed other Ensemble algorithms recently [67]. XGBoost is extremely fast due to the parallelization of trees with improved performance through algorithmic enhancements. However, a major drawback of XGBoost is the lower classification performance for imbalanced data. In this study, an extensive experiment was done to show that our proposed model outperformed these models in terms of detecting PD patients. In this work, for mathematical analysis, Python 3.7 was used as the programming platform. A powerful computer was used for fast data processing with the following configuration: Intel^®^ Core™ i7-8665UE Processor, 16 GB RAM, 1 TB Hard disk, etc.

## 5. Results and Analysis

The proposed models were used to detect PD patients. For the experiment, the dataset mentioned in Section 3 was used to evaluate the model performance to find out the best one, and comparisons with MLP, KNN, RF, and XGBoost were exhibited. The dataset was split into 70% and 30% for training and test purposes, respectively, with no subject occurring in the test or train data simultaneously. In this study, the features stated in Table 2 were taken as input, and the classification label was given as output. The range of (0–1) was used for the scaling purpose of the original dataset and normalization.

The output of our classifier can be evaluated in terms of accuracy. However, the dataset used in this study had class imbalance with a ratio of 188:64, and so measuring only the accuracy can be misleading in terms of evaluating the classifiers. Therefore, the results of the classifier were measured in terms of the accuracy, F1-score, Mathews Correlation Coefficient (MCC), and Precision-Recall curves. Accuracy can be defined as:

(15) $$Accuracy=\frac{Number\;of\;correct\;predictions}{Total\;number\;of\;predictions\;made}$$

To calculate the F1-score, MCC, and Precision-Recall curve, a confusion matrix is expressed in Table 3 to understand the binary classifier predictions with *tp*, *fp*, *tn*, and *fn* as true positive, false positive, true negative, and false negative, respectively.

From the confusion matrix, the F1-score can be defined as:

(16) $$Precision=\frac{tp}{tp+fp}$$

(17) $$Recall=\frac{tp}{tp+fn}$$

(18) $$F1-score=\frac{2\times Precision\times Recall}{Precision+Recall}$$

MCC can be defined as:

(19) $$MCC=\frac{tp\times tn-fp\times fn}{\sqrt{\left(tp+fp\right)\left(tp-fp\right)\left(tn+fp\right)\left(tn+fn\right)}}$$

**Figure 6:**
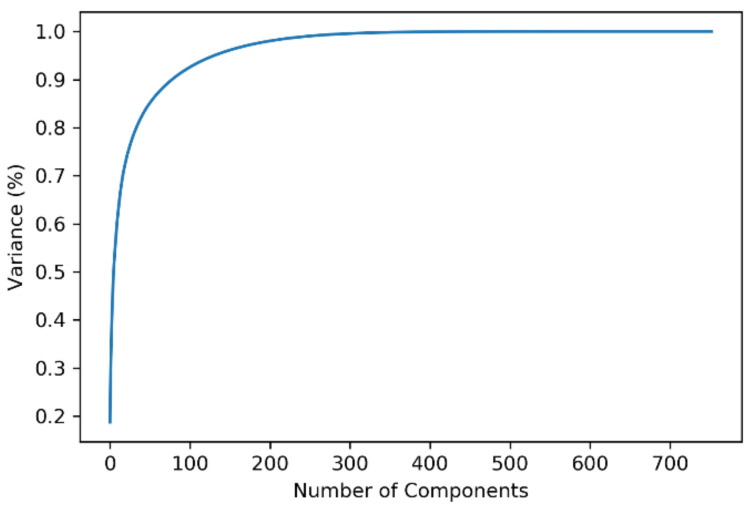
Parkinson’s disease dataset explained variance.

In our study, the polynomial kernel function was used with degree 2 for the SVM, the gamma was set to 10 for the non-linear hyperplane, and C was set to 1 to avoid both under-fitting and over-fitting. The best output was obtained from this optimal set of hyper-parameters via k-fold cross-validation, where k was set to 10 for modest variance and low bias with a mean of 0.8 and a standard deviation of 0.06 (approx.). For the first model, including PCA, from Figure 6, it can be implied that the number of principal components needed to be set to 200 to preserve around 98.8–99% of the total variance of the data and reduce components with insignificant variance from the input. Table 4 bolsters this intuition of selecting 200 principal components compared with other variable numbers of principal components, fed to the SVM classifier, as it showed the highest accuracy, F1-score, and MCC value of 0.889, 0.928, and 0.7, respectively. Without reducing the number of principal components, and with only the removal of feature redundancy, the training time was 0.203 s, with an accuracy, F1-score, and MCC score of 0.885, 0.925, and 0.688, respectively; whereas with the reduced 200 principal components, the training time was 0.022 s with higher accuracy, F1-score, and MCC score of 0.889, 0.928, and 0.7 respectively.

**Figure 7:**
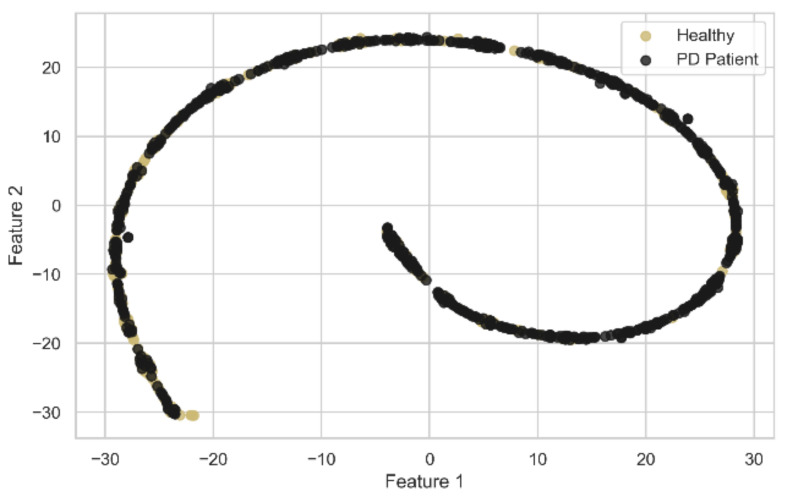
2-D representation of data points using t-SNE before encoding with SAE.

**Figure 8:**
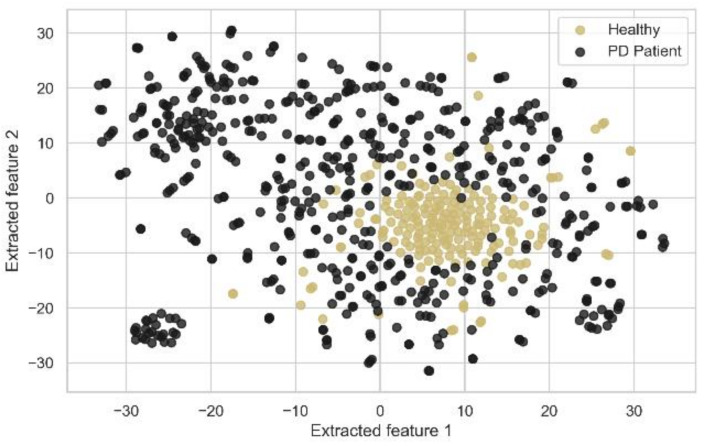
2-D representation of data points using t-SNE after encoding with SAE.

To evaluate the second model, including the novel deeper SAE with five dense encoding layers with tanh activation and L1 regularization, using adam as the optimizer, *MSE* as the loss function, a batch size of 32, and integrated with SVM, the actual raw data points are plotted in Figure 7 with the two most important features after using t-SNE [64]. We initialized t-SNE with dimensions 2, perplexity 50, seed 24,680, and random state 0. t-SNE is used to learn, investigate, or evaluate segmentation. It can be employed to visualize the clear data separation. Figure 7 depicts that the data points were not easily separable, and the features had a non-linear relationship with each other. After applying SAE to the raw data points and feeding the extracted features to the SVM classifier, the best performance was gained by setting the extracted feature number to 180 with comparisons with other feature numbers, as shown in Table 5. Figure 8 also shows the data points with the two most important features by using t-SNE, as mentioned earlier, among the extracted 180 features by the SAE. In Figure 8, the data points are easily separable from the data points in Figure 7, and it can be seen that the segmentation actually holds up. Juxtaposing Table 4 and Table 5, the SAE-SVM model outperformed the former PCA-SVM model with the highest accuracy of 0.935, the highest F1-score of 0.951, and the highest MCC value of 0.788. The non-linear relationship between the vocal features of the PD patients resulted in the better performance of the SAE-SVM model, which outperformed the former PCA-SVM model as PCA features are linearly uncorrelated. Therefore, the deep neural SAE with multiple hidden layers was more successful in compressing the information into a lower-dimensional feature space.

The MLP, XGBoost, KNN, and RF classifiers were implemented to evaluate the effectiveness of the models in this study. The prediction of PD was made with all the models providing the same set of data. From the experimental result, it can be seen that KNN showed the least efficiency among all the models and XGBoost had a better performance than others, but our proposed SAE-SVM model outperformed all the other models in terms of accuracy, F1-score, and MCC. Table 6 states the accuracy, F1-score, and MCC value of all the models tested and shows that the proposed SAE-SVM model had the highest accuracy of 0.935, the highest F1-score of 0.951, and the highest MCC value of 0.788. It also shows the performance boost of SVM with dimensionality reduction, which in the case of the SAE-SVM was a 9.5% increase in accuracy, 4.9% increase in F1-score, and 32% increase in MCC score compared to the SVM. In addition, it can be inferred that the SAE-SVM had the lowest misdiagnosis rate with 6.5%, and the PCA-SVM had the second lowest misdiagnosis rate with 11.1%.

**Figure 9:**
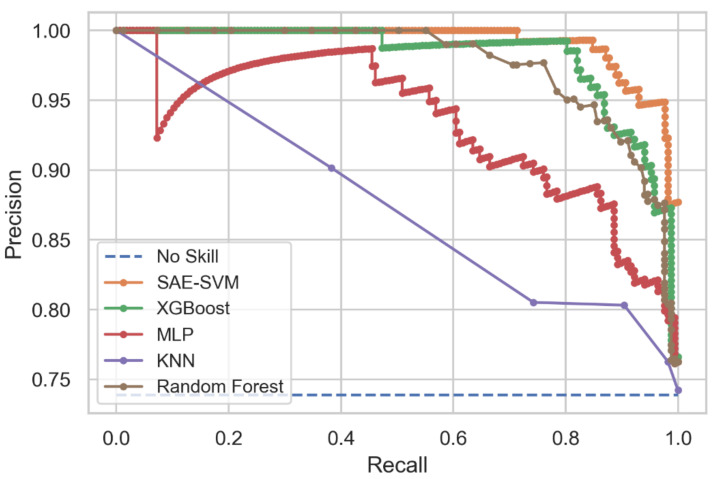
Precision-Recall curve comparison among all the models.

Figure 9 illustrates the Precision-Recall curve from the experimental results. It depicts that the KNN model had the lowest AUC value with 0.875 and that XGBoost showed a higher AUC of 0.981, but the proposed SAE-SVM model surpassed both models with an AUC of 0.988.

We also compared our best model with other commonly used feature extraction techniques, including the LDA and ICA implementing and incorporating with the same SVM model. Table 7 shows that our best model of novel deeper SAE with SVM performed better in terms of accuracy, F1-score, and MCC value than the LDA and ICA, as our deeper SAE was more efficient in reducing the dimensionality of feature space and improving the network’s generalization property.

Along with our implemented models, we compared our result with two recent studies made by Sakar et al. [16], in which an SVM using RBF kernel gave the best result, and Gunduz [24], in which the CNN framework had the best output. We compared the results of our model with their best results, as they used the same dataset. From Table 8, it can be seen that our proposed SAE-SVM model outperformed both studies in terms of accuracy, F1-score, and MCC value.

We also used SMOTE for oversampling. From Table 9, our models showed better accuracy, F1 score, and MCC value in the balanced-class scenario.

## 6. Discussion

In this study regarding the detection of PD patients from vocal signals, we depicted and implemented two models based on two different feature extraction algorithms along with SVM, which is a popular supervised algorithm in the area of classification problems, using hyperplanes to classify both linear and non-linear dataset. In the first model, a PCA was used as it is a popular unsupervised method for finding the principal components of data in order to reduce the dimensions. This, in turn, bypassed the disadvantage of SVM with decreasing classification performance while having a higher number of features than the number of samples as in the dataset used in this study. In the second model, a novel deeper SAE was developed and used for the same purpose, which is a DNN and works in an unsupervised manner to map the features to a new feature space, reducing the curse of dimensionality problem.

The above-mentioned models were trained and tested using the dataset obtained from the University of California-Irvine (UCI) Machine Learning repository. Due to the high imbalance in the dataset, the F1-score, MCC, and Precision-Recall curve were used to evaluate the models along with accuracy.

For the evaluation of models, all the feature sets, including baseline features, time-frequency features, MFCCs, Wavelet Transform-based features, vocal fold features, and TQWT, were concatenated for the feature extraction purpose. For the first model, 200 principal components were selected from the explained variance of the dataset. Later these 200 principal components were fed into the SVM classifier with a polynomial kernel of degree 2, and it was compared against other variable numbers of principal components. The highest accuracy, F1-score, and MCC were acquired when the number of principal components was set to 200. For the second model, the novel deeper SAE converted the features into 180 features which also distributed the data points in a more easily separable fashion with the polynomial SVM. Other variable numbers of features were also tested, and the results were not as satisfactory as the 180 extracted features. From both the models, it can be implied that the SAE-SVM showed an increase in accuracy rate by 5% (approx.) than that of the PCA-SVM. This was also seen in the F1-score and MCC rate as they increased from 0.928 to 0.951 and 0.7 to 0.788, respectively. Moreover, according to the present study, the best model (SAE-SVM) outperformed other models, including XGBoost, MLP, KNN, and RF. It also outperformed the SVM with other common feature extraction techniques, LDA and ICA. Moreover, it was successful at surpassing the two most recent works of Sakar et al. [16] and Gunduz [24] in terms of the accuracy, F1-score, and MCC metrics, and had the lowest misdiagnosis rate of 6.5%. It can be concluded that our proposed SAE-SVM model is a good alternative to both of the models proposed in these two literatures.

The salient advantages of the proposed best model of this study over the previous PD detection studies are stated below:

- Different feature extraction techniques were applied, and the relative comparisons were depicted with a much larger dataset with 752 features and 756 voice samples, unlike the recent study in Hemmerling and Sztahó [28], which has only 198 voice samples and 33 features. Small datasets have several disadvantages, which lead to lower precision in the prediction, lower power, and pose a larger risk by comparing the classes unfairly, even in the circumstances that the data is from a randomized trial. They used PCA only to remove feature redundancy; in contrast, in this study, PCA was used to reduce dimensionality as well as remove feature redundancy, which boosted the training time efficiency and all the performance metrics of our model for a larger dataset.
- In our study, the non-linear relationship between vocal features of the PD patients was depicted in Figure 7. For these non-linear feature relations, our second model SAE-SVM outperformed the former PCA-SVM model as PCA works well with linear relationships. Therefore, the novel deep neural SAE with multiple hidden layers was more successful in compressing the information better into a lower-dimensional feature space. Deep feature extraction of SAE augmented the discriminatory power of distinguishing PD patients as established by the increased MCC value.
- As in other previous literatures, if accuracy was used as the only evaluation metric, it can be misleading in the case of an imbalanced dataset. However, inspired by Sakar et al. [16], we used F1-score and MCC along with accuracy. We also used the Precision-Recall curve to visualize the performance of the models for such skewed class distribution. We also applied SMOTE to synthesize new minority examples to evaluate the models in a balanced-class scenario. Both models showed better balanced-class performance.

The study explored the field of Parkinson’s disease patient detection based on vocal features by building the idea of merging feature extractions, removal of irrelevant data by reducing dimensionality based on the variance of data and additionally using DNN in an unsupervised manner with SVM, which is one of the most powerful classifiers thus far when it comes to data points separable with a larger number of hyperplanes. Imbalanced data deters from having an accurate picture in the detection of PD patients, which can be solved with a more balanced dataset. Further research and experiments can be conducted by employing other dimensionality reduction and feature extraction algorithms such as kernel PCA (kPCA), Denoising Autoencoders to reduce the noise effects of voice signals, etc. The performance of the model can also be improved by applying enhancement algorithms to reduce reverberation, background noise and non-linear distortion [68,69]. Along with these, the performance of the proposed model can be further improved with the inclusion of wearable sensor data for measuring tremors and postural instability of individuals to detect the PD features more accurately.

## Figures and Tables

**Table 1:** Details of the dataset.

Data Set Characteristic	Multivariate
Attribute characteristic	Integer, Real
Total attributes	754
Total instances	756
Subjects involved	252
Healthy individuals involved	64 (23 men and 41 women) with an average age of 61.5 years
PD patient involved	188 (107 men and 81 women) with an average age of 60 years
Types of classification	Binary (healthy (class 0), PD patient (class 1))
Voice recordings per person	3
Missing values	N/A
Data Collection Process	Microphone frequency = 44.1 KHz. Each individual sustained phonation of the vowel/a/was collected by following the physician’s examination
Generated by	Department of Neurology in Cerrahpasa Faculty of Medicine, Istanbul University with the approval of the Clinical Research Ethics Committee of Bahcesehir University

**Table 2:** Feature sets of Parkinson’s disease data [24].

Feature	Measure	Number of Features
Baseline features	Jitter variants	5
	Shimmer variants	6
	Fundamental frequency parameters	5
	Harmonicity parameters	2
	Recurrence period density entropy (RPDE)	1
	Detrended fluctuation analysis (DFA)	1
	Pitch period entropy (PPE)	1
Time-frequency features	Intensity parameters	3
	Formant frequencies	4
	Bandwidth	4
Mel-frequency cepstral coefficients (MFCCs)	MFCCS	84
Wavelet transform-based features	Wavelet transform-based features	182
Vocal fold features	Glottis quotient (GQ)	3
	Glottal to noise excitation (GNE)	6
	Vocal fold excitation ratio (VFER)	7
	Empirical mode decomposition (EMD)	6
Tunable Q-factor wavelet transform (TQWT)	TQWT features	432

**Table 3:** Confusion matrix for binary classification.

	PD Predicted	Healthy Predicted
**PD actual**	*tp*	*fn*
**Healthy actual**	*fp*	*tn*

**Table 4:** Performance comparisons with variable principal components.

# of Principal Components	Accuracy	F1-Score	MCC
100	0.876	0.919	0.675
150	0.885	0.925	0.688
180	0.887	0.926	0.695
200	0.889	0.928	0.7
230	0.885	0.925	0.688

**Table 5:** Performance comparisons with variable feature numbers of SAE.

# of Principal Components	Accuracy	F1-Score	MCC
100	0.845	0.901	0.62
150	0.898	0.935	0.71
180	0.935	0.951	0.788
200	0.912	0.943	0.737
230	0.888	0.921	0.689

**Table 6:** Performance comparisons with traditional ML models.

Model	Accuracy	F1-Score	MCC
RF	0.836	0.897	0.539
MLP	0.845	0.897	0.602
KNN	0.765	0.851	0.325
XGBoost	0.881	0.924	0.676
SVM	0.854	0.907	0.595
PCA-SVM	0.889	0.928	0.7
SAE-SVM	0.935	0.951	0.788

**Table 7:** Comparison with other feature extraction techniques with the SVM classifier.

Model	Accuracy	F1-Score	MCC
LDA	0.513	0.593	0.079
ICA	0.819	0.888	0.480
Deeper SAE	0.935	0.951	0.788

**Table 8:** Comparison with previous works.

Model	Accuracy	F1-Score	MCC
SVM (RBF)	0.86	0.84	0.59
CNN framework	0.869	0.917	0.632
SAE-SVM	0.935	0.951	0.788

**Table 9:** Performance with the balanced dataset.

Model	Accuracy	F1-Score	MCC
PCA-SVM	0.894	0.91	0.71
SAE-SVM	0.944	0.964	0.83
